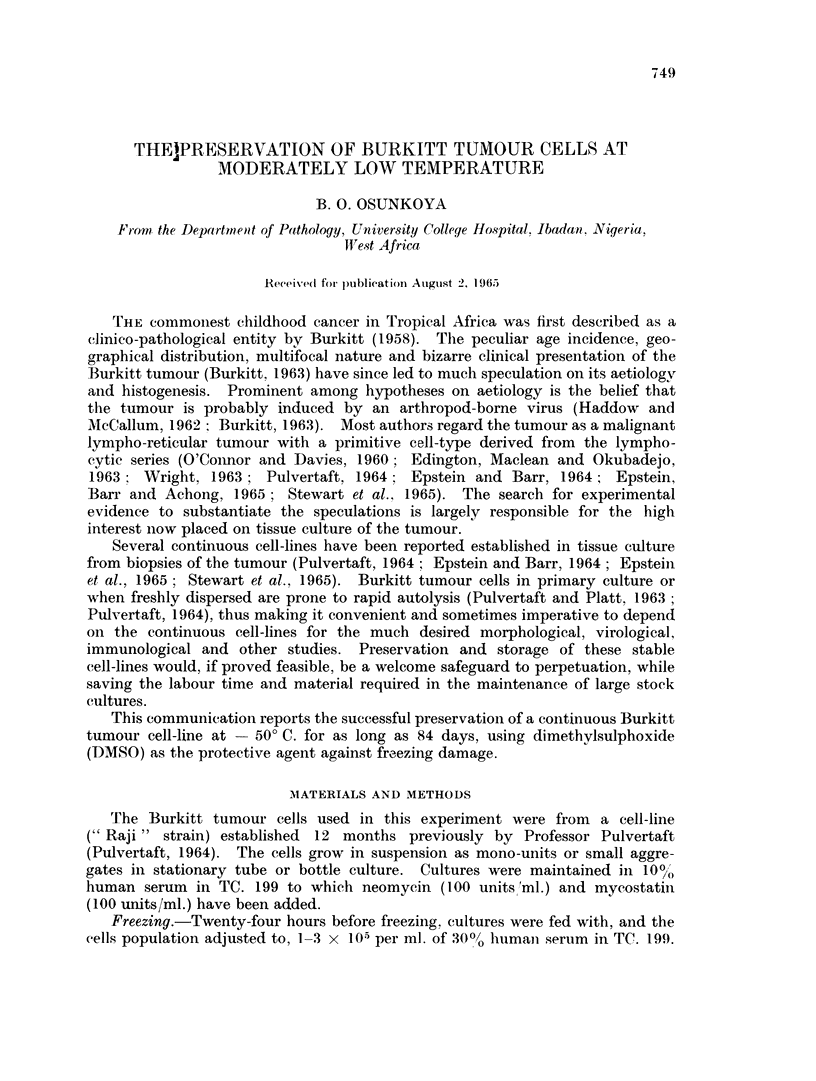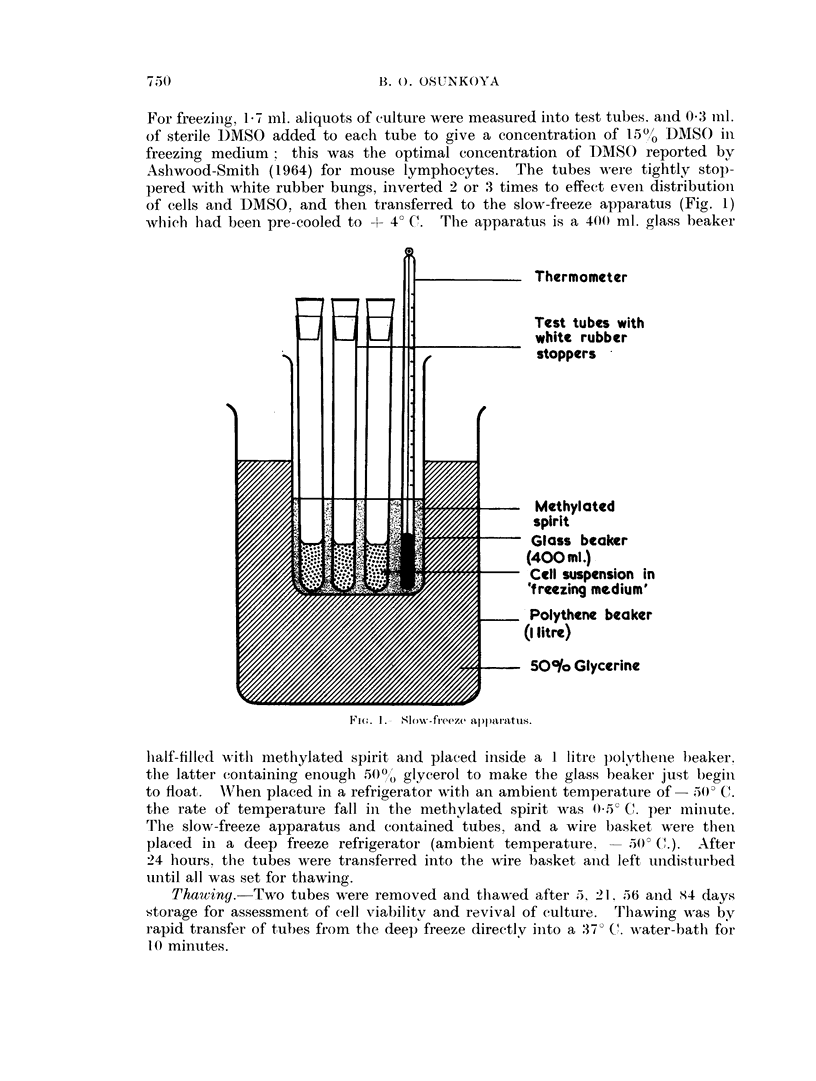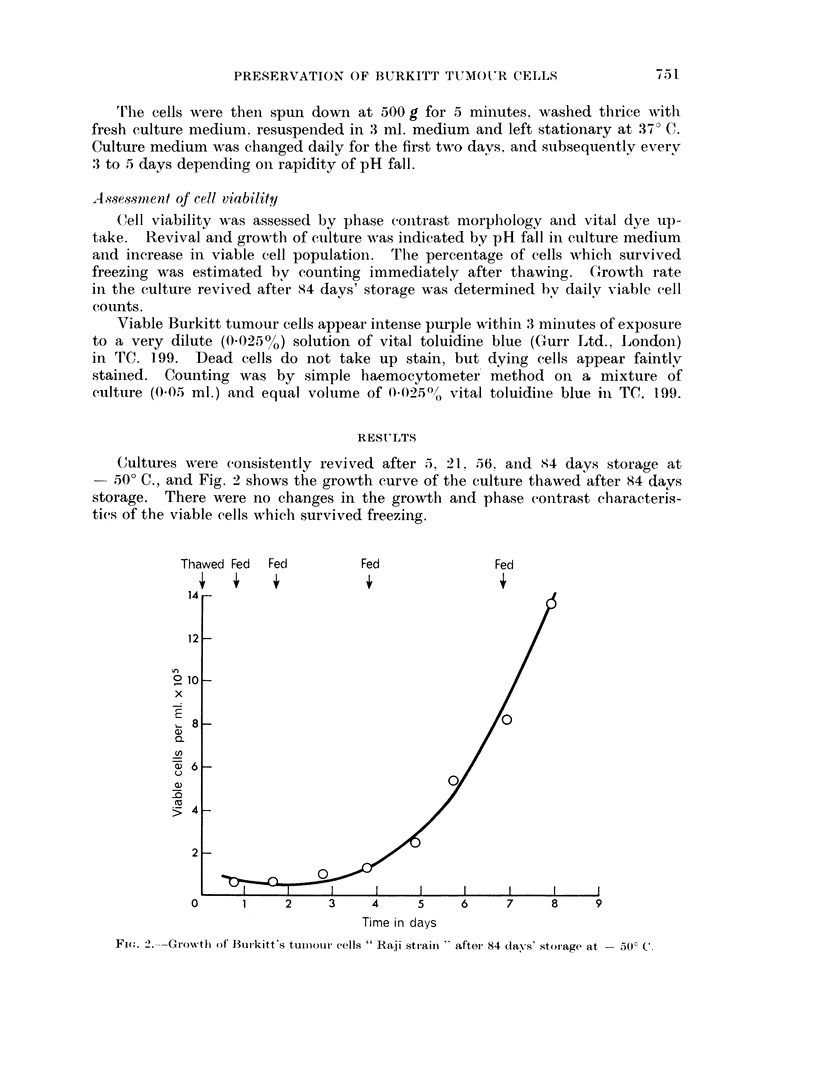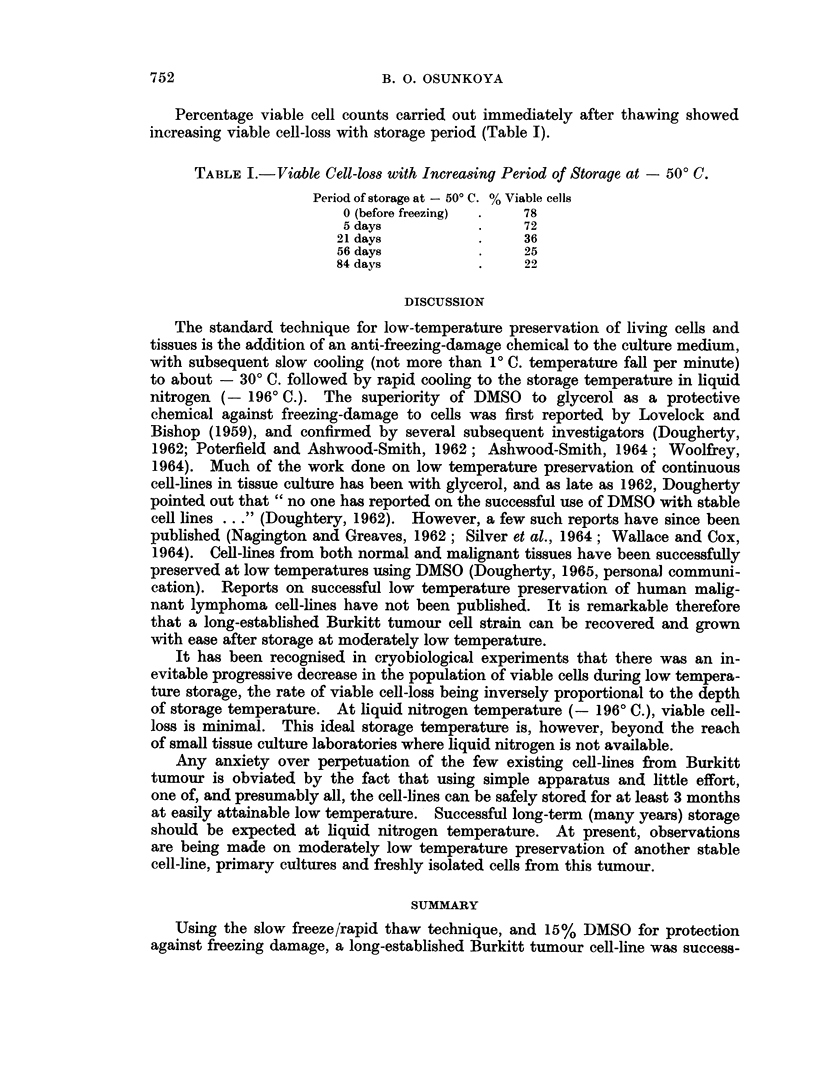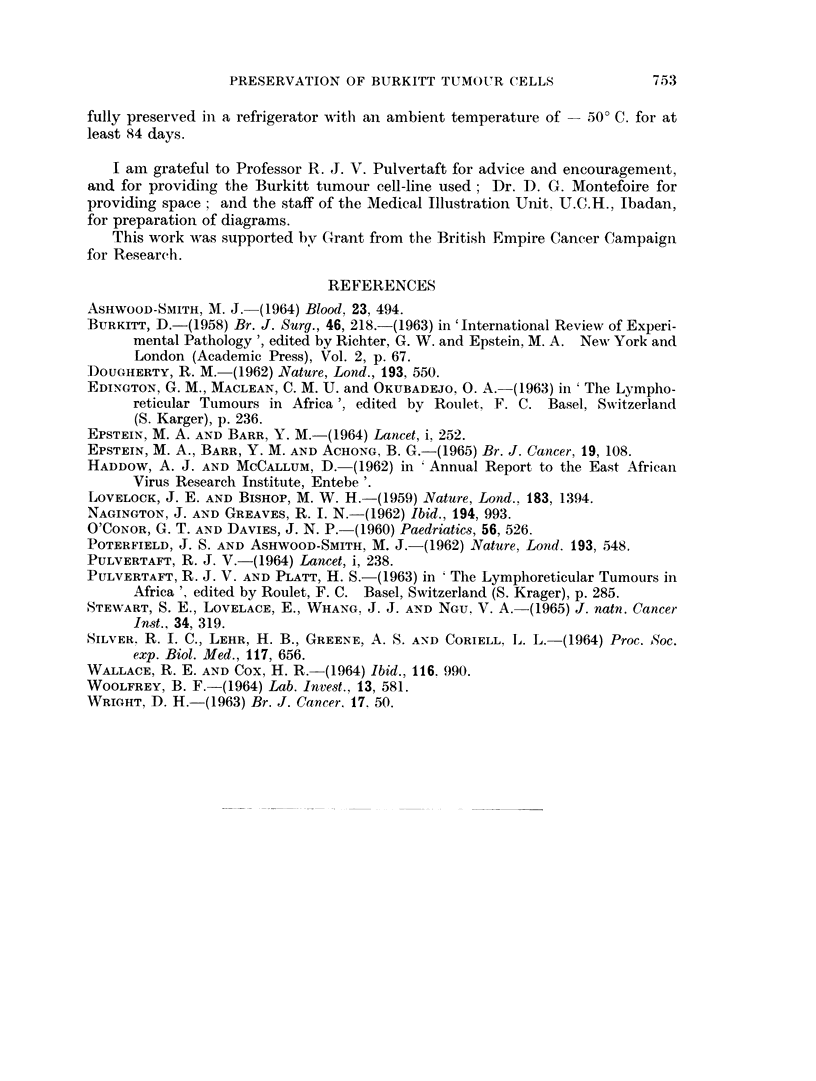# The preservation of Burkitt tumour cells at moderately low temperature.

**DOI:** 10.1038/bjc.1965.87

**Published:** 1965-12

**Authors:** B. O. Osunkoya


### THE PRESERVATION OF BURKITT TUMOUR CELLS AT MODERATELY LOW TEMPERATURE

B. O. OSUNKOYA

*From the Department of Pathology, University College Hospital, Ibadan, Nigeria, West Africa*

Received for publication August 2, 1965

THE commonest childhood cancer in Tropical Africa was first described as a clinico-pathological entity by Burkitt (1958). The peculiar age incidence, geographical distribution, multifocal nature and bizarre clinical presentation of the Burkitt tumour (Burkitt, 1963) have since led to much speculation on its aetiology and histogenesis. Prominent among hypotheses on aetiology is the belief that the tumour is probably induced by an arthropod-borne virus (Haddow and McCallum, 1962 ; Burkitt, 1963). Most authors regard the tumour as a malignant lympho-reticular tumour with a primitive cell-type derived from the lymphocytic series (O'Connor and Davies, 1960 ; Edington, Maclean and Okubadejo, 1963 ; Wright, 1963 ; Pulvertaft, 1964 ; Epstein and Barr, 1964 ; Epstein, Barr and Achong, 1965 ; Stewart *et al.*, 1965). The search for experimental evidence to substantiate the speculations is largely responsible for the high interest now placed on tissue culture of the tumour.

Several continuous cell-lines have been reported established in tissue culture from biopsies of the tumour (Pulvertaft, 1964 ; Epstein and Barr, 1964 ; Epstein *et al.*, 1965 ; Stewart *et al.*, 1965). Burkitt tumour cells in primary culture or when freshly dispersed are prone to rapid autolysis (Pulvertaft and Platt, 1963 ; Pulvertaft, 1964), thus making it convenient and sometimes imperative to depend on the continuous cell-lines for the much desired morphological, virological, immunological and other studies. Preservation and storage of these stable cell-lines would, if proved feasible, be a welcome safeguard to perpetuation, while saving the labour time and material required in the maintenance of large stock cultures.

This communication reports the successful preservation of a continuous Burkitt tumour cell-line at − 50° C. for as long as 84 days, using dimethylsulphoxide (DMSO) as the protective agent against freezing damage.

### MATERIALS AND METHODS

The Burkitt tumour cells used in this experiment were from a cell-line (" Raji " strain) established 12 months previously by Professor Pulvertaft (Pulvertaft, 1964). The cells grow in suspension as mono-units or small aggregates in stationary tube or bottle culture. Cultures were maintained in 10% human serum in TC. 199 to which neomycin (100 units/ml.) and mycostatin (100 units/ml.) have been added.

*Freezing.*—Twenty-four hours before freezing, cultures were fed with, and the cells population adjusted to, 1–3 × $10^5$ per ml. of 30% human serum in TC. 199.

For freezing, 1·7 ml. aliquots of culture were measured into test tubes, and 0·3 ml. of sterile DMSO added to each tube to give a concentration of 15% DMSO in freezing medium; this was the optimal concentration of DMSO reported by Ashwood-Smith (1964) for mouse lymphocytes. The tubes were tightly stoppered with white rubber bungs, inverted 2 or 3 times to effect even distribution of cells and DMSO, and then transferred to the slow-freeze apparatus (Fig. 1) which had been pre-cooled to + 4° C. The apparatus is a 400 ml. glass beaker

FIG. 1.—Slow-freeze apparatus.

half-filled with methylated spirit and placed inside a 1 litre polythene beaker, the latter containing enough 50% glycerol to make the glass beaker just begin to float. When placed in a refrigerator with an ambient temperature of — 50° C. the rate of temperature fall in the methylated spirit was 0·5° C. per minute. The slow-freeze apparatus and contained tubes, and a wire basket were then placed in a deep freeze refrigerator (ambient temperature, — 50° C.). After 24 hours, the tubes were transferred into the wire basket and left undisturbed until all was set for thawing.

*Thawing.*—Two tubes were removed and thawed after 5, 21, 56 and 84 days storage for assessment of cell viability and revival of culture. Thawing was by rapid transfer of tubes from the deep freeze directly into a 37° C. water-bath for 10 minutes.

The cells were then spun down at 500 *g* for 5 minutes, washed thrice with fresh culture medium, resuspended in 3 ml. medium and left stationary at 37° C. Culture medium was changed daily for the first two days, and subsequently every 3 to 5 days depending on rapidity of pH fall.

*Assessment of cell viability*

Cell viability was assessed by phase contrast morphology and vital dye uptake. Revival and growth of culture was indicated by pH fall in culture medium and increase in viable cell population. The percentage of cells which survived freezing was estimated by counting immediately after thawing. Growth rate in the culture revived after 84 days' storage was determined by daily viable cell counts.

Viable Burkitt tumour cells appear intense purple within 3 minutes of exposure to a very dilute (0·025%) solution of vital toluidine blue (Gurr Ltd., London) in TC. 199. Dead cells do not take up stain, but dying cells appear faintly stained. Counting was by simple haemocytometer method on a mixture of culture (0·05 ml.) and equal volume of 0·025% vital toluidine blue in TC. 199.

### RESULTS

Cultures were consistently revived after 5, 21, 56, and 84 days storage at — 50° C., and Fig. 2 shows the growth curve of the culture thawed after 84 days storage. There were no changes in the growth and phase contrast characteristics of the viable cells which survived freezing.

FIG. 2.—Growth of Burkitt's tumour cells " Raji strain " after 84 days' storage at — 50° C.

Percentage viable cell counts carried out immediately after thawing showed increasing viable cell-loss with storage period (Table I).

TABLE I.—*Viable Cell-loss with Increasing Period of Storage at — 50° C.*

| Period of storage at — 50° C. | % Viable cells |
|---|---|
| 0 (before freezing) | 78 |
| 5 days | 72 |
| 21 days | 36 |
| 56 days | 25 |
| 84 days | 22 |

### DISCUSSION

The standard technique for low-temperature preservation of living cells and tissues is the addition of an anti-freezing-damage chemical to the culture medium, with subsequent slow cooling (not more than 1° C. temperature fall per minute) to about — 30° C. followed by rapid cooling to the storage temperature in liquid nitrogen (— 196° C.). The superiority of DMSO to glycerol as a protective chemical against freezing-damage to cells was first reported by Lovelock and Bishop (1959), and confirmed by several subsequent investigators (Dougherty, 1962; Poterfield and Ashwood-Smith, 1962 ; Ashwood-Smith, 1964 ; Woolfrey, 1964). Much of the work done on low temperature preservation of continuous cell-lines in tissue culture has been with glycerol, and as late as 1962, Dougherty pointed out that " no one has reported on the successful use of DMSO with stable cell lines . . ." (Doughtery, 1962). However, a few such reports have since been published (Nagington and Greaves, 1962 ; Silver *et al.*, 1964 ; Wallace and Cox, 1964). Cell-lines from both normal and malignant tissues have been successfully preserved at low temperatures using DMSO (Dougherty, 1965, personal communication). Reports on successful low temperature preservation of human malignant lymphoma cell-lines have not been published. It is remarkable therefore that a long-established Burkitt tumour cell strain can be recovered and grown with ease after storage at moderately low temperature.

It has been recognised in cryobiological experiments that there was an inevitable progressive decrease in the population of viable cells during low temperature storage, the rate of viable cell-loss being inversely proportional to the depth of storage temperature. At liquid nitrogen temperature (— 196° C.), viable cell-loss is minimal. This ideal storage temperature is, however, beyond the reach of small tissue culture laboratories where liquid nitrogen is not available.

Any anxiety over perpetuation of the few existing cell-lines from Burkitt tumour is obviated by the fact that using simple apparatus and little effort, one of, and presumably all, the cell-lines can be safely stored for at least 3 months at easily attainable low temperature. Successful long-term (many years) storage should be expected at liquid nitrogen temperature. At present, observations are being made on moderately low temperature preservation of another stable cell-line, primary cultures and freshly isolated cells from this tumour.

### SUMMARY

Using the slow freeze/rapid thaw technique, and 15% DMSO for protection against freezing damage, a long-established Burkitt tumour cell-line was successfully preserved in a refrigerator with an ambient temperature of — 50° C. for at least 84 days.

I am grateful to Professor R. J. V. Pulvertaft for advice and encouragement, and for providing the Burkitt tumour cell-line used ; Dr. D. G. Montefoire for providing space ; and the staff of the Medical Illustration Unit, U.C.H., Ibadan, for preparation of diagrams.

This work was supported by Grant from the British Empire Cancer Campaign for Research.

### REFERENCES

ASHWOOD-SMITH, M. J.—(1964) *Blood*, **23**, 494.

BURKITT, D.—(1958) *Br. J. Surg.*, **46**, 218.—(1963) in 'International Review of Experimental Pathology ', edited by Richter, G. W. and Epstein, M. A. New York and London (Academic Press), Vol. 2, p. 67.

DOUGHERTY, R. M.—(1962) *Nature, Lond.*, **193**, 550.

EDINGTON, G. M., MACLEAN, C. M. U. and OKUBADEJO, O. A.—(1963) in ' The Lymphoreticular Tumours in Africa ', edited by Roulet, F. C. Basel, Switzerland (S. Karger), p. 236.

EPSTEIN, M. A. AND BARR, Y. M.—(1964) *Lancet*, i, 252.

EPSTEIN, M. A., BARR, Y. M. AND ACHONG, B. G.—(1965) *Br. J. Cancer*, **19**, 108.

HADDOW, A. J. AND McCALLUM, D.—(1962) in ' Annual Report to the East African Virus Research Institute, Entebe '.

LOVELOCK, J. E. AND BISHOP, M. W. H.—(1959) *Nature, Lond.*, **183**, 1394.

NAGINGTON, J. AND GREAVES, R. I. N.—(1962) *Ibid.*, **194**, 993.

O'CONOR, G. T. AND DAVIES, J. N. P.—(1960) *Paedriatics*, **56**, 526.

POTERFIELD, J. S. AND ASHWOOD-SMITH, M. J.—(1962) *Nature, Lond.* **193**, 548.

PULVERTAFT, R. J. V.—(1964) *Lancet*, i, 238.

PULVERTAFT, R. J. V. AND PLATT, H. S.—(1963) in ' The Lymphoreticular Tumours in Africa ', edited by Roulet, F. C. Basel, Switzerland (S. Krager), p. 285.

STEWART, S. E., LOVELACE, E., WHANG, J. J. AND NGU, V. A.—(1965) *J. natn. Cancer Inst.*, **34**, 319.

SILVER, R. I. C., LEHR, H. B., GREENE, A. S. AND CORIELL, L. L.—(1964) *Proc. Soc. exp. Biol. Med.*, **117**, 656.

WALLACE, R. E. AND COX, H. R.—(1964) *Ibid.*, **116**, 990.

WOOLFREY, B. F.—(1964) *Lab. Invest.*, **13**, 581.

WRIGHT, D. H.—(1963) *Br. J. Cancer*, **17**, 50.